# Processing and Shelf-Life Prediction Models for Ready-to-Eat Crayfish

**DOI:** 10.3390/foods14081296

**Published:** 2025-04-08

**Authors:** Qian Li, Jieyu Lei, Keying Su, Xiaoying Chen, Laihoong Cheng, Chunmin Yang, Shiyi Ou

**Affiliations:** 1 School of Engineering, Guangzhou College of Technology and Business, Guangzhou 510850, China; liqian23@student.usm.my (Q.L.); lilyquinn9406@gmail.com (J.L.); sukeying@student.usm.my (K.S.); 15975728290@163.com (X.C.); 2 Food Technology Division, School of Industrial Technology, Universiti Sains Malaysia (USM), Gelugor 11800, Malaysia; lhcheng@usm.my; 3 Department of Food and Engineering, Jinan University, Guangzhou 510632, China

**Keywords:** crayfish, storage period, quantity, shelf-life, kinetic model

## Abstract

This study investigated the production process of ready-to-eat crayfish, focusing on changes in sensory quality, pH, total volatile base nitrogen (TVB-N), total viable count (TVC), acid value (AV), springiness, and hardness during storage at 4 °C, 25 °C, and 37 °C. A shelf-life prediction model was developed using the Arrhenius model. The optimal crayfish formula was determined to be 0.12% spices, 0.80% salt, and a stewing time of 70 min, which achieved the highest sensory score of 9.25 points. This combination resulted in shrimp meat with an intact texture, a soft and smooth taste, and rich spicy and briny flavors. A Pearson correlation analysis showed significant correlations among TVB-N, TVC, AV, springiness, and hardness. When fitting each indicator with zero-order, first-order, and second-order kinetics, TVB-N, AV, and springiness aligned more closely with the zero-order kinetics model, while TVC and hardness fit better with the first-order kinetics model. The Arrhenius equation-based shelf-life model demonstrated an error margin of 9.1% between predicted and actual quality indicators, confirming its feasibility for predicting the quality and shelf life of spicy crayfish. These findings provide a crucial theoretical basis for the intelligent prediction of storage and distribution conditions for ready-to-eat crayfish.

## 1. Introduction

Predicting food shelf-life is essential for producers, distributors, and consumers, as it helps determine storage duration and optimal consumption timing [1]. Accurate shelf-life predictions rely on scientific methodologies integrating microbiological, sensory, and biochemical indicators during storage, often coupled with mathematical models such as the Arrhenius model, neural network model, random forest model, and partial least squares method [2,3,4]. Perishable foods are highly sensitive to external ambient temperatures, making precise temperature control essential for reducing spoilage rates and extending shelf life. However, evaluating shelf life at lower temperatures requires longer assessment periods [5]. Consequently, using higher storage temperatures alongside kinetic reaction rates and the Arrhenius model can expedite the testing periods. The combined use of kinetic reaction and the Arrhenius model is widely adopted for predicting the shelf life of fruits, vegetables, meats, and aquatic products, among others [6]. For instance, Zhang et al., 2021 [7] utilized a kinetic model to forecast the storage duration of kiwifruit, while Dabadé et al., 2023 [8] and Don et al., 2018 [9] employed the Arrhenius model to establish a predictive model for the shelf life of tropical brackish shrimp.

Crayfish are small crustaceans that inhabit freshwater bodies such as rivers, streams and lakes, and are widely consumed globally. As of 2023, China is the world’s largest crayfish producer, with an annual output of approximately 2.5 million tons [10]. Crayfish are a valuable source of high-quality protein, essential amino acids, and various vitamins and minerals [11]. Major consumer markets include China, the United States, and Europe, where crayfish are enjoyed in diverse settings such as night markets, chain restaurants, and home cooking. They are particularly popular among younger consumers, who tend to consume them more frequently [12,13]. Due to perishability after death, the prompt handling of large crayfish harvests is crucial. Converting crayfish into ready-to-eat products offers a solution to overproduction, which constitutes one-third of the total production [14,15]. The processing of ready-to-eat food typically involves several key steps: raw material selection and cleaning, cooking, seasoning and braising, cooling, packaging, transportation, and storage, which are all aimed at preserving flavor, quality, and food safety [16,17].

Due to perishability after death, prompt handling of large crayfish harvests is crucial.

This study aims to optimize the preparation process of ready-to-eat crayfish by examining changes in sensory quality, pH levels, TVB-N, TVC, AV, springiness, and hardness across different storage temperatures. Additionally, it analyzes the interrelationships among these parameters and develops a mathematical model for predicting the shelf life of ready-to-eat crayfish. The study objectives include: (1) exploring the processing of ready-to-eat crayfish; (2) examining the quality changes in ready-to-eat crayfish under different storage temperatures; (3) evaluating the effectiveness of various kinetic models in describing the quality transformations of spicy crayfish; and (4) predicting the quality changes and shelf life of spicy crayfish at different storage temperatures.

## 2. Materials and Methods

### 2.1. Materials and Reagents

Crayfish was purchased from a local market (Shayuan Market, Guangzhou, China) with an average weight of 25 ± 1 g. Crayfish were transported to the laboratory on ice within 30 min of collection. Cumin, cardamom, allspice, cinnamon, star anise, cinnamon, grass nuts, angelica dahurica, cloves, sambucus, licorice, dried peppercorns, dried chili peppers, ginger, salt, and edible oil were purchased from the same local market. Haday seasoning wine was purchased from Foshan Haitian Seasoned Food Co. (Foshan, China), Seasoned Food Co. (Foshan, China), and Chill oil (main ingredient soybean oil) was purchased from Lee Kum Kee Foods Co. (Shanghai, China) Potassium hydrogen phthalate, potassium dihydrogen phosphate, sodium dihydrogen phosphate, magnesium oxide, and potassium hydroxide were purchased from Sinopharm Group Chemical Reagent Co. (Shanghai, China) Plate counting agar (PCA) was purchased from Guangdong Huankai Microbiology Co. (Guangzhou, China).

### 2.2. Ready-to-Eat Crayfish Typical Production Process

The frozen crayfish were first thawed and then rinsed under running tap water for 5 min to remove surface debris. They were subsequently decontaminated by immersion in a 5% sodium chloride solution for 10 min.

For cooking, 3 kg of water was mixed with 12–48 g of a spice package, maintaining the following ratio: cumin, Chinese cinnamon, cardamom, sesame leaf, dried peppercorns, and dried chili peppers (3 parts each); cinnamon, star anise, licorice, cloves, angelica dahurica, and grass nuts (1 part each). The mixture was brought to a boil at atmospheric pressure and maintained for 30 min. Add 100 g of crayfish to the boiling water along with 30 g of ginger, 15 g of Haday seasoning wine, 13 g of chicken bouillon and 10.5–28.5 g of salt. Cook at 100 °C for 15 min under atmospheric pressure. Then, reduce the heat to a temperature of 85 °C and continue cooking for an additional 50–90 min to enhance flavor absorption and texture development.

At the end of the cooking process, the crayfish were removed from the stock and transferred to 304 stainless steel tubs. They were then cooled in an ice water bath until their temperature reached 25 °C within 30 min. After cooling, 4 g of chili oil and 16 g of crayfish soup base were added and mixed thoroughly, ensuring a crayfish–marinating liquid ratio of approximately 5:1 (*w*/*w*). The marinated crayfish were then placed in sterilized, food-grade plastic ziplock bags and vacuum-sealed at −0.08 Mpa.

### 2.3. Single-Factor Test of the Ready-to-Eat Crayfish Production Process

In the experimental design, preliminary data from the crayfish preparation process indicated that proportionally varying the levels of spice and salt contributes to maintaining consistent flavor. Specifically, a balanced proportion of spices ensured an optimal overall flavor, enhancing the sensory qualities of the crayfish without overwhelming the dish with either spice or salt. Initial testing of the basic recipe outlined in Section 2.2, with all other parameters held constant, revealed that the flavor was well-balanced when the spice amount was set at 24 g (0.8% spice concentration), with an equal amount of salt added (24 g, 0.8% salt concentration), and a simmering time of 60 min. Based on these findings, the effects of varying spice concentrations (0.6%, 0.8%, 1.0%, 1.2%, 1.4%), salt additions (0.35%, 0.50%, 0.65%, 0.80%, 0.95%), and simmering times (50 min, 60 min, 70 min, 80 min, and 90 min) on the organoleptic qualities of ready-to-eat crayfish were further investigated to optimize the process.

### 2.4. Sensory Evaluation

Sensory characteristics were evaluated by an experienced panel of 20 individuals (ten males and ten females). Panelists rated the samples on a 10-point scale ranging from 1 to 10 for color, appearance, flavor, taste, and texture (1 = very disliked to 10 = very good). During storage, sensory quality was assessed based on color, appearance, flavor, and texture. The overall sensory score for each sample was calculated as the average of these parameters. Scores below 5 were considered unacceptable [18].

### 2.5. Analysis on Physicochemical Properties of Ready-to-Eat Crayfish

#### 2.5.1. Sample Processing

The ready-to-eat crayfish, prepared under the optimal cooking conditions determined from the single-factor test, were vacuum-packaged and divided into three groups for storage at 4 °C, 25 °C, and 37 °C. Samples that were stored at 4 °C were tested on days 0, 2, 4, 6, 8, and 10. Samples stored at 25 °C and 37 °C were tested at hours 0, 12, 24, 36, 48, and 60.

#### 2.5.2. Determination of pH

Five grams of ready-to-eat crayfish samples were blended (PT 1300 D, BUCHI, Flawil, Switzerland) homogeneously with 10 mL of distilled water. The pH value of the samples was measured using a digital pH meter (S400-Micro, METTLER TOLEDO, Greifensee, Switzerland) at 25 °C [19].

#### 2.5.3. Determination of TVC

TVC was determined according to the Chinese National Standard Method for Food Safety, Microbiological Determination of Food (2022) [20]. Approximately, 25 g of crayfish samples were weighed into a sterile homogenizing bag containing 225 mL of sterile saline and tapped with a tapping homogenizer for 2 min, followed by the addition of sterile saline to prepare a 10-fold series of dilutions of the sample homogenate (10^−1^ to 10^−9^). Then, an aliquot of 1 mL of the diluted sample was inoculated into the plate counting agar and incubated at 30 °C for 72 h. The results of TVC were recorded in log CFU/g.

#### 2.5.4. Determination of Total Volatile Base Nitrogen (TVB-N)

TVB-N was determined according to the China National Standard Method for Food Safety, Determination of Total Volatile Base Nitrogen in Food (2016) [21]. Briefly, 10 g of crayfish samples were weighed into a distillation tube with 75 mL of water. The samples were macerated for 30 min, before adding 1 g of magnesium oxide and being mixed homogeneously. After that, samples were analyzed using a fully automated Kjeldahl nitrogen analyzer (K1100, Hanon, Jinan, China), and the results were expressed as mg/100 g, and each test was repeated for three times.

#### 2.5.5. Determination of Acid Value (AV)

The acid value (AV) of ready-to-eat crayfish was determined using hot ethanol titration method, following the guidelines of the China National Standard Method for Food Safety (2016) [22]. Approximately 10 g of stirred crayfish samples were weighed into a 250 mL conical flask, before adding 100 mL of neutralized ethanol solution and heating to a boil in a water bath. During boiling, the conical flask was shaken vigorously to form a suspension, removed, and then titrated with 0.1 mol/L KOH standard solution. The AV was calculated according to the following equation:

X_AV_ = (V − V_0_) × c × 56.1/m, (1)

where V is the volume of standard titration solution consumed by the specimen determination (mL); V_0_ is the volume of standard titration solution consumed by the blank determination (mL); c is the molar concentration of standard titration solution (mol/L); 56.1 is the molar mass of potassium hydroxide (g/mol); and m is the weight of the sample (g).

#### 2.5.6. Determination of Hardness and Elasticity

Crayfish meat pieces, approximately 1 cm × 1 cm × 0.7 cm in size, were analyzed for elasticity and hardness using a Brookfield CT3 texture analyzer (Brookfield Engineering Laboratories, INC. Middleboro, MA, USA). The analysis employed a TA10 probe with testing parameters of 2 s test interval, 180 mm/min test rate, 50% deformation, and 12 mm distance between the probe and sample after lifting. Each sample was examined with 10 replicates. TexturePro CT V1.9 professional software was used to determine hardness and springiness of the test samples.

### 2.6. Kinetic Models Based on Freshness Index

Kinetic models were fitted with TVB-N, TVC, AV, springiness, and hardness for ready-to-eat crayfish at 4, 25, and 37 °C. The oxidation kinetic models were analyzed using the equations shown in the following zero-, first-, and second-order equations [23]:

Zero order model: k = (A − A_0_)/t, (2)

First order model: k = (lnA − lnA_0_)/t, (3)

Second order model: k = (1/A − 1/A_0_)/t, (4)

where A_0_ represents the initial value, and A denotes the freshness index value at any storage time t (days).

### 2.7. Prediction of Shelf Life

The temperature dependence of the reaction rate constant (k) was obtained using the Arrhenius model, which is represented by the following equation:

lnk = lnk_0_ − Ea/RT. (5)

Taking logarithms on both sides gives the following equation:

k = k_0_exp(−Ea/RT). (6)

The integration of kinetic methods into shelf-life equations to generate predictive models for ready-to-eat crayfish is represented by the following equations:

Zero-order:SL = (A − A_0_)/k, (7)

First-order:SL = (lnA − lnA_0_)/k, (8)

where k_0_ is the reaction rate (constant) at the reference temperature, Ea (KJ mol^−1^), T (K), and R (J K^−1^ mol^−1^) are the activation energy, the absolute temperature and the molar gas constant 8.3144 J K^−1^ mol^−1^, respectively. SL is the storage time of ready-to-eat crayfish, A0 is the initial value, and A is the value of the freshness index at any storage time t (d).

### 2.8. Data Analysis

The data were analyzed using analysis of variance (ANOVA) in SPSS 27 (IBM, New York City, NY, USA), with significance level set at *p* < 0.05. Each dataset comprised three measurements, and results are presented as mean ± standard deviation. Graphical representation was conducted using Origin 2022. The fitted model quality was assessed based on the correlation coefficient (R^2^).

## 3. Results

### 3.1. Optimization of the Preparation Process of Ready-to-Eat Crayfish

In this experiment, the selection of spice addition, salt content, and stewing duration for the one-way test was based on their significant impact on the flavor profile of ready-to-eat crayfish. Salt plays a pivotal role in culinary applications by enhancing taste and contributing to flavor development. Additionally, an extended stewing period results in a richer concentration of aromatic compounds, intensifying overall aroma [24]. However, excessive stewing duration can lead to shrimp meat dispersion and loss of elasticity.

Table 1 illustrates that the level of 0.12% spice addition, 0.80% salt content, and 70 min of stewing time yielded the highest sensory score of 9.25 points. Under these conditions, the crayfish exhibited a rich aroma, with shrimp meat remaining intact, devoid of any signs of degradation, and maintaining optimal elasticity [25].

### 3.2. Changes in Physicochemical Indicators of Ready-to-Eat Crayfish Under Different Storage Conditions

#### 3.2.1. Changes in Total Viable Count

The proliferation and growth of microorganisms are the primary factors contributing to food spoilage, with crayfish’s high protein content providing an ideal medium for microbial development. Stewing at elevated temperatures effectively eliminated microorganisms present in raw crayfish. Following cooking, the initial total viable count (TVC) of crayfish was recorded at 0.57 log CFU/g, consistently remaining below 1 log CFU/g. This observation is supported by findings from Martínez-Álvarez et al., 2009 [1].

**Figure 1:**
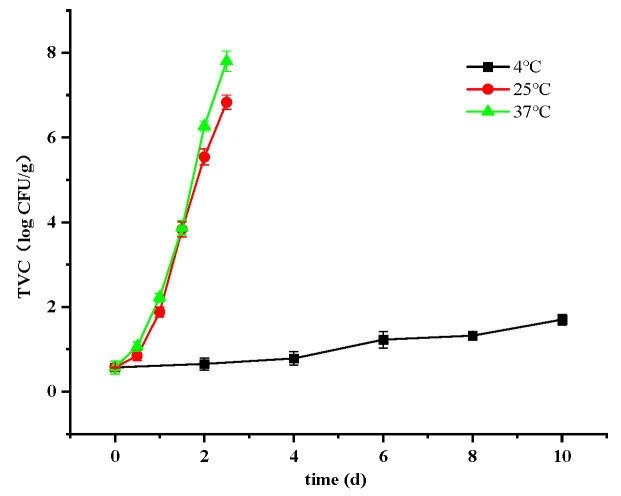
Changes in TVC values of ready-to-eat crayfish at different storage temperatures. Error bars represent standard error from three measurements.

The rate of microbial growth increased significantly with higher storage temperatures. By the end of the study period, TVCs for crayfish stored at 4 °C, 25 °C, and 37 °C reached 1.7, 6.83, and 7.8 log CFU/g, respectively (Figure 1). It is noteworthy that acceptable microbiological criteria for cooked meat products typically require counts below 6 log CFU/g [24]. Accordingly, all TVCs remained within acceptable limits at 4 °C throughout the storage period. For crayfish stored at 25 °C and 37 °C, permissible storage durations based on TVC considerations were determined as 2 and 1.5 days, respectively.

#### 3.2.2. Changes in pH, TVB-N, and AV

**Figure 2:**
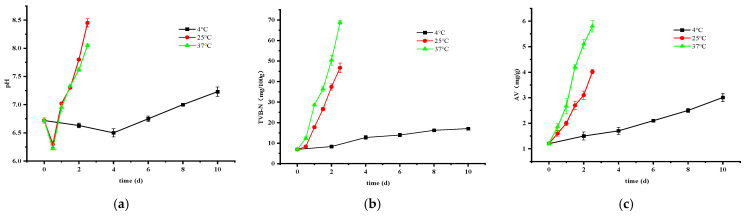
Changes in pH (**a**), TVB-N (**b**), and AV (**c**) at different storage temperatures. Error bars represent standard error from three measurements.

The impact of storage temperature on pH (Figure 2a), total volatile basic nitrogen (TVB-N) (Figure 2b), and acid value (AV) (Figure 2c) was significant, with higher temperatures accelerating increases in these parameters. Initially, pH declined during early storage phases due to the accumulation of carbohydrates like glycogen, which undergo glycolytic conversion to acids, especially lactic acid, within crayfish muscle tissue [26]. As storage time progresses and the total viable count of microorganisms increases, leading to the degradation of more proteins and the subsequent accumulation of nitrogenous alkaline compounds, such as amino acids, trimethylamine, and indoles. These compounds further interact with organic acids, promoting the formation of higher levels of volatile basic nitrogen compounds, ultimately resulting in an elevated pH and TVB-N levels [27].

TVB-N levels (Figure 2b) correlate closely with the degree of spoilage in animal-derived foods. The acceptable threshold for TVB-N is typically 35 mg/100 g [28]. Accordingly, permissible storage durations under safe conditions were determined as 1.5 days at 25 °C and 1 day at 37 °C based on TVB-N considerations.

Additionally, the hydrolysis and oxidation of fats and oils in food products contribute to rancidity and deterioration, directly impacting quality and safety. An acceptable acid value (AV) is generally ≤5.0 mg KOH/g [29]. By the end of the study period, AV levels remained within acceptable limits at 4 °C and 25 °C. However, storage at 37 °C for 1.5 days resulted in an acceptable AV. It is worth noting that although spoilage was detected after 2 days at 25 °C, AV did not exceed the standard. This could be attributed to crayfish’s relatively low-fat content, which helps mitigate AV elevation beyond acceptable levels.

#### 3.2.3. Changes in Springiness and Hardness

The combined impact of endogenous and exogenous factors contributes to a decline in the hardness and springiness of crayfish, attributed to phenomena such as water loss, microbial proliferation, and eventual protein depletion. Elevated temperatures promote increased microbial growth and reproduction, further accelerating protein degradation.

**Figure 3:**
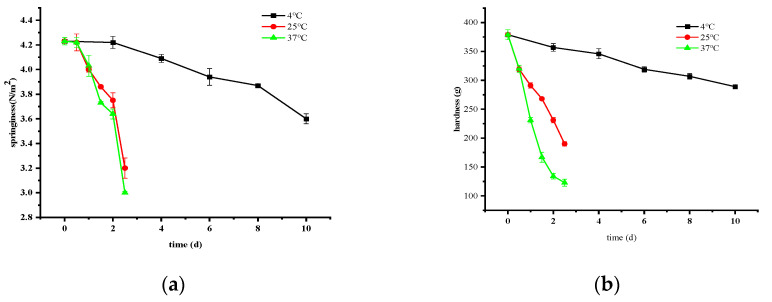
Changes in springiness (**a**) and hardness (**b**) of ready-to-eat crayfish at different storage temperatures. Error bars represent standard error from three measurements.

Figure 3 illustrates that the initial springiness and hardness of ready-to-eat crayfish were recorded as 4.23 N/m^2^ and 379 g, respectively. Over the storage durations at 4 °C, 25 °C, and 37 °C until the conclusion of the study period, springiness values decreased to 3.6 N/m^2^, 3.2 N/m^2^, and 3 N/m^2^, respectively. Correspondingly, hardness exhibited reductions of 23.75%, 49.87%, and 67.55%, respectively.

### 3.3. Changes in Sensory Quality of Ready-to-Eat Crayfish Under Different Storage Conditions

**Figure 4:**
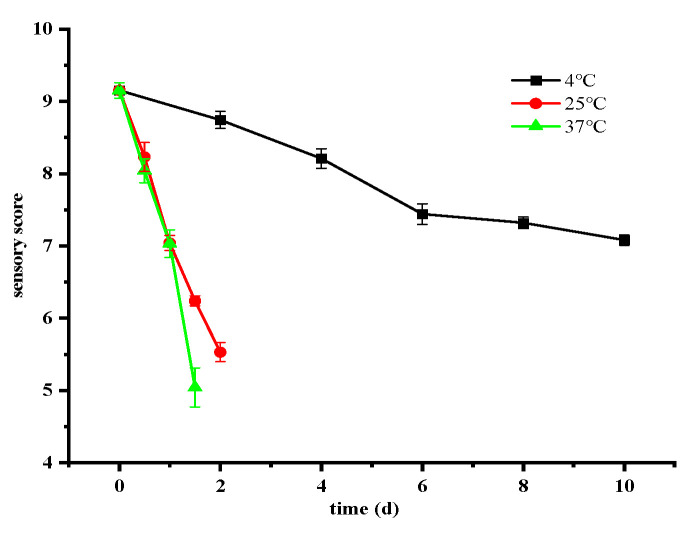
Changes in sensory quality of ready-to-eat crayfish at different storage temperatures. Error bars represent standard error from three measurements.

The changes in sensory quality during storage are depicted in Figure 4. With prolonged storage duration, a noticeable decline in sensory quality was observed, particularly at higher storage temperatures. A critical threshold was identified when the average sensory score fell below 5, indicating unacceptable levels and rendering the product unfit for consumption. However, considering the safety of ready-to-eat crayfish, the critical values of TVC and TVB-N were used as tasting endpoints, thus allowing a storage time of 2 days at 25 °C and 1.5 days at 37 °C. In contrast, at 4 °C, the sensory quality score remained consistently high at 7.08 throughout the study period, indicating permissible continued storage.

### 3.4. Correlation Analysis of Each Freshness Index Under Different Storage Conditions

**Figure 5:**
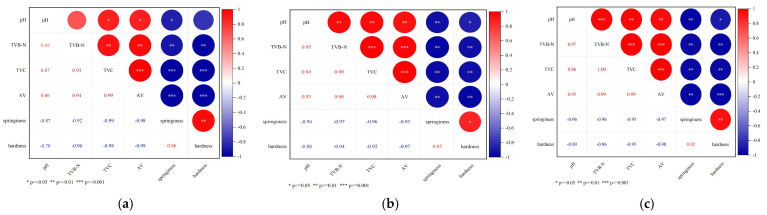
Pearson correlation of freshness indices of ready-to-eat crayfish products analyzed at 4 °C (**a**), 25 °C (**b**) and 37 °C (**c**). * *p* ≤ 0.05, ** *p* ≤ 0.01, *** *p* ≤ 0.001.

Throughout the storage period of ready-to-eat crayfish, significant changes were observed between physicochemical properties, revealing a notable correlation among the indices understudied. The degree of correlation, assessed using Pearson’s coefficient, indicates a strong association between the variables, as depicted in Figure 5.

At 4 °C, correlation coefficients between total viable count (TVC), total volatile basic nitrogen (TVB-N), acid value (AV), hardness, and springiness all exceeded 0.9. In contrast, the correlation with pH was found to be insignificant (*p* ≥ 0.05). However, at 25 °C and 37 °C, the sensory score exhibited significant correlations with each freshness index (*p* < 0.05). Specifically, pH, TVB-N, TVC, and AV were positively correlated with sensory score, and the reverse is true for hardness and springiness. Based on this comprehensive correlation analysis, TVB-N, TVC, AV, hardness, and springiness indices were selected as key factors in developing a kinetics prediction model for shelf-life assessment.

### 3.5. Determination of Kinetic Parameters and Reaction Constants

Table 2 presents the R^2^ coefficients and RMSE values of zero-level, one-level, and two-level response models for quality indicators. A higher R^2^ coefficient signifies a stronger linear relationship and greater fitting accuracy [30]. The results demonstrate that the quality indicators of ready-to-eat crayfish exhibited better fitting with zero-order and first-order response models, with R^2^ values all exceeding 0.87. Notably, the ΣR^2^ values of the zero-order response model for TVB, AV, and elasticity surpassed those of the first-order response model, recording values of 2.906, 2.945, and 2.694, respectively. Moreover, the ΣR^2^ values of the first-order response model for TVB and hardness were notably higher, standing at 2.888 and 2.958, respectively.

The RMSE values illustrate model accuracy, with lower values indicating better fit. For TVB-N and AV, the zero-order model had the lowest RMSE at lower temperatures (0.967 and 0.107 at 4 °C) but increased with temperature, reducing accuracy. Springiness followed a similar trend. TVC had relatively low RMSE across models, suggesting good fit. Hardness showed the most variation, with zero-order RMSE peaking at 28.032 at 37 °C, indicating poor accuracy. Overall, zero- and first-order models performed better than the second-order model, but accuracy declined at higher temperatures.

Consequently, the zero-order kinetic model was selected to characterize the changes in TVB, AV, and springiness of crayfish. Furthermore, the first-order kinetics model was chosen to delineate the changes in TVC and hardness.

### 3.6. Establishment of Arrhenius Equation for Quality Indicators of Ready-to-Eat Crayfish

As indicated in Table 3, the natural logarithm of the reaction rate constant (ln k) exhibits a linear relationship with the reciprocal of temperature (1/T). The reaction rate constants (k) for various metrics were determined at temperatures of 4 °C, 25 °C, and 37 °C, and subsequently fitted linearly. Notably, the reaction rate constants (k) for springiness and hardness are of negative values; thus, their absolute values were used to derive the appropriate regression equations. Consequently, regression equations were derived, yielding pre-factor values (K_0_) and activation energy (Ea). These values were integrated into Equations (7) and (8), resulting in the formulation of kinetic shelf-life prediction models for TVB-N, TVC, AV, springiness, and hardness across varying storage temperatures.

Upon examining Table 4, it is evident that each index exhibited a high degree of fit (R^2^ > 0.898), indicating the model’s adeptness in accurately predicting the time-dependent changes in the quality indices of ready-to-eat crayfish across diverse storage temperatures.

### 3.7. Validation and Evaluation of Shelf-Life Prediction Models

As depicted in Table 5, the relative standard deviation between the predicted and measured values of the shelf-life prediction model, established using TVB-N, TVC, AV, springiness, and hardness of ready-to-eat crayfish as indicators at a storage temperature of 10 °C, all fall within 9.5%. This close alignment between the actual and predicted shelf-life of ready-to-eat crayfish attests to the model’s high level of credibility. Table 5 lists the predicted and measured quality indicators for ready-to-eat crayfish.

## 4. Conclusions

Crayfish is a highly popular delicacy in China and various Southeast Asian countries. Understanding its processing and storage characteristics, along with developing a shelf-life model for industrial use, is important. Higher temperatures accelerate changes in sensory quality, physical and chemical indices, and texture, making the fish more prone to spoilage and deterioration. Key oxidation indices such as TVB-N, TVC, AV, hardness, and springiness were analyzed. A shelf-life prediction model was created using a kinetic model combined with the Arrhenius equation. The model showed high accuracy and relevance for TVB-N, AV, and hardness when using zero-order reaction kinetics, while TVC and springiness were best fitted with first-order reaction kinetics. The prediction model demonstrated excellent accuracy and correlation, with relative errors between predicted and measured values remaining below 9.5%. This validates the reliability and effectiveness of the Arrhenius equation-based model for predicting the shelf-life of ready-to-eat crayfish, facilitating improved industrial processing.

## Figures and Tables

**Table 1:** Results of single-factor test on the production process of ready-to-eat crayfish.

Spice Additions (%)	Score	Salt Addition (%)	Score	Simmering Time (min)	Score
0.6	7.62 ± 0.08 ^c^	0.35	7.91 ± 0.09 ^d^	50	7.52 ± 0.11 ^d^
0.8	7.83 ± 0.06 ^c^	0.50	8.13 ± 0.06 ^c^	60	8.29 ± 0.07 ^b^
1.0	8.36 ± 0.10 ^b^	0.65	8.37 ± 0.06 ^b^	70	9.25 ± 0.10 ^a^
1.2	8.92 ± 0.09 ^a^	0.80	8.96 ± 0.09 ^a^	80	8.28 ± 0.12 ^b^
1.4	8.46 ± 0.05 ^b^	0.95	8.06 ± 0.12 ^cd^	90	7.80 ± 0.10 ^c^

The values presented are the mean ± standard error (*n* = 3). Distinct letters denote statistically significant differences among samples (*p* < 0.05).

**Table 2:** Kinetic modeling parameters for quality changes in ready-to-eat crayfish at different storage temperatures.

Indicators	Temperature (°C)	Zero-Order			First-Order			Second-Order		
		R^2^	RSEM	∑R^2^	R^2^	RSEM	∑R^2^	R^2^	RSEM	∑R^2^
TVB-N	4	0.956	0.967	2.906	0.910	1.382	2.856	0.873	1.492	2.556
	25	0.972	2.961		0.972	2.955		0.886	4.019	
	37	0.978	3.848		0.974	4.208		0.797	4.025	
TVC	4	0.952	0.108	2.878	0.970	0.084	2.888	0.960	0.105	2.625
	25	0.963	0.551		0.952	0.534		0.855	1.278	
	37	0.963	0.629		0.966	0.600		0.810	2.301	
AV	4	0.980	0.107	2.945	0.998	0.032	2.94	0.977	0.031	2.705
	25	0.979	0.170		0.993	0.100		0.856	1.160	
	37	0.986	0.248		0.949	0.467		0.872	1.802	
Springiness	4	0.934	0.070	2.694	0.924	0.077	2.639	0.913	0.505	2.554
	25	0.876	0.151		0.856	0.161		0.821	0.813	
	37	0.884	0.178		0.859	0.195		0.820	1.017	
Hardness	4	0.988	3.630	2.916	0.991	3.578	2.958	0.989	5.463	2.919
	25	0.982	10.052		0.983	9.829		0.952	20.864	
	37	0.943	28.032		0.984	14.868		0.978	32.768	

**Table 3:** Results of fitting Arrhenius’ equation for different temperatures to the reaction rate constant k for the quality indicator of ready-to-eat crayfish.

Indicator	Kinetic Grade	Simultaneous Equations	R^2^	RSEM	Activation Energy Ea/(KJ·mol^−1^)	Predisposing Factors K_0_
TVB-N	Zero-level	lnK = 30.935 − 8507.390/T	0.949	0.313	70.736	2.72 × 10^13^
TVC	First-order	lnK =17.924 − 5527.722/T	0.898	0.298	45.961	6.09 × 10^7^
AV	Zero-level	lnK = 19.695 − 5911.062/T	0.968	0.172	49.149	3.58 × 10^8^
springiness	Zero-level	lnK = 17.259 − 5521.926/T	0.939	0.224	45.913	4.10 × 10^7^
Hardness	First-order	lnK = 24.527 − 7776.532/T	0.988	0.137	64.660	4.49 × 10^10^

**Table 4:** Shelf-life prediction model for quality indicators of ready-to-eat crayfish.

Indicator	Arrhenius Model
TVB-N	SL_TVB-N_ = (A − A_0_)/[2.72×10^13^ exp (−8695.909/T)]
TVC	SL_TVC =_ (lnA − lnA_0_)/[6.09×10^7^ exp (−5650.202/T)]
AV	SL_AV_ = (A − A_0_)/[3.58×10^8^ exp (−6042.117/T)]
Springiness	SL_S_ = (A − A_0_)/[4.10×10^7^ exp (−5644.301/T)]
Hardness	SL_H_ = ln(A − A_0_)/[4.49×10^10^ exp (−77,948.958/T)]

**Table 5:** Predicted and measured shelf-life of ready-to-eat crayfish.

Quality Indicators	Storage Time (day)	Measured Value	Predicted Value	Relative Standard Deviation (%)	RSEM
TVB-N (mg/100 g)	2	12.71	11.79	7.80	
	4	17.18	16.57	3.66	
	6	21.09	21.36	1.27	0.97
	8	27.00	26.15	3.25	
	10	29.31	30.94	5.24	
TVC (log CFU/g)	2	0.81	0.84	4.33	
	4	1.21	1.26	4.22	
	6	2.04	1.88	8.80	0.18
	8	2.76	2.81	1.79	
	10	3.83	4.19	8.45	
AV (mg/g)	2	1.95	1.81	7.62	
	4	2.51	2.42	3.89	
	6	3.05	3.02	0.87	0.16
	8	3.42	3.63	5.78	
	10	4.48	4.24	5.61	
Springiness (N/m^2^)	2	3.76	3.96	4.99	
	4	3.73	3.68	1.43	
	6	3.16	3.40	7.14	0.15
	8	3.21	3.13	2.58	
	10	2.94	2.85	3.11	
Hardness (g)	2	324.27	341.38	5.01	
	4	335.13	307.49	8.99	
	6	251.84	276.96	9.07	21.06
	8	268.20	249.47	7.51	
	10	238.05	224.70	5.94	

## Data Availability

The original contributions presented in the study are included in the article, further inquiries can be directed to the corresponding authors.

## Abbreviations

The following abbreviations are used in this manuscript:

TVB-N	Total volatile base nitrogen
TVB-N	Total viable count
AV	Acid value
